# Myocarditis and Rhabdomyolysis in a Healthy Young Man Caused by Salmonella Gastroenteritis

**DOI:** 10.1155/2015/954905

**Published:** 2015-06-01

**Authors:** Warkaa Al Shamkhani, Yasmeen Ajaz, Nagham Saeed Jafar, Sunil Roy Narayanan

**Affiliations:** Department of Internal Medicine & Cardiology, Belhoul Speciality Hospital, Dubai, UAE

## Abstract

Salmonella gastroenteritis is a common, self-limiting, foodborne disease and a rare cause of life-threatening complications especially in immunocompetent individuals. Moreover, bacterial infections of the GI tract have been rarely reported as a cause of serious complications like acute myocarditis and rhabdomyolysis. While viral infections are commonly associated with myocarditis, bacterial infections are infrequently seen with these conditions. Similarly, bacterial infections may lead to only 5% of adult rhabdomyolysis events whereas viral-induced myositis appears to be the commonest. A 28-year-old young male with no past medical problems presented with acute salmonella gastroenteritis that was complicated by myocardial injury (most likely myocarditis), rhabdomyolysis, acute renal failure, and shock. He made an uneventful complete recovery of all complications by early recognition of these rare complications and prompt institution of appropriate therapy.

## 1. Introduction

*Salmonella enteritidis* is one of the commonest serotypes of salmonella bacteria reported globally, especially in underdeveloped countries. Eggs and poultry have been largely linked as a source of food contaminated with salmonella. Acute gastroenteritis is the commonest presentation. The course of the illness usually follows a benign course of 4–7 days that usually resolves without the need of antibiotics. Complications like osteomyelitis are infrequently seen in a patient with good immunity. Acute rhabdomyolysis, myocarditis, acute renal failure, and shock are very rare [1, 2].

Elderly, infants and immunocompromised patients may develop a more severe illness with spread to other body sites that can lead to death if not treated with prompt antibiotics. Acute myocarditis and rhabdomyolysis are two life-threatening conditions that can be caused by infective, toxic, or autoimmune etiologies. Myocarditis presents with various presentations ranging from nonspecific chest pain to cardiogenic shock. The clinical sequel of myocarditis includes hypovolemia, metabolic acidosis, hyperkalemia, acute kidney injury, and disseminated intravascular coagulation [3].

In very rare occasions, salmonella infections may present in an unusual way in immunocompetent individuals. A high index of suspicion is needed to early recognize serious complications. Chest pain or ECG changes, severe muscle pain, or weakness in a very ill-looking patient with diarrhea should be promptly investigated.

## 2. Case Report

A 28-year-old male presented to the emergency department with severe fatigue and inability to move his limbs since 6 hours. He had a history of severe, nonbloody diarrhea (10–20 times/day) with frequent vomiting since 2 days. On examination, he was severely dehydrated, with a heart rate of 130 beats per minute, blood pressure of 80/50 mmHg, and temperature of 38.6°C. There were severe muscle weakness and muscle tenderness. Abdomen examination showed a superficial generalized tenderness.

**Figure 1:**
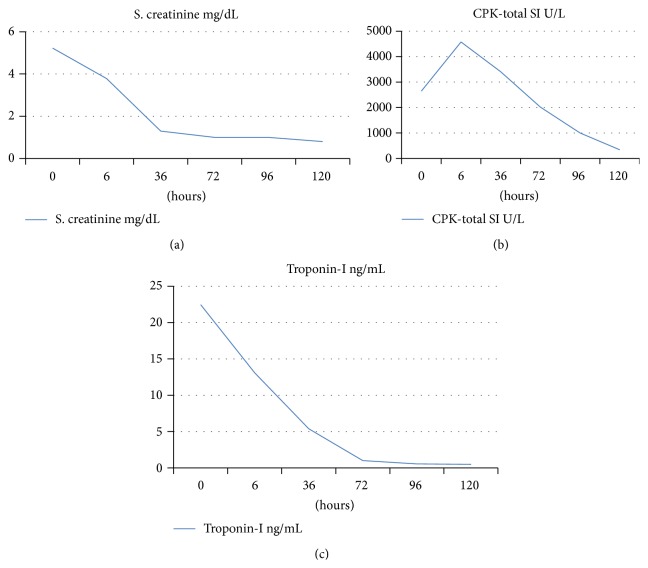
Line graph illustrating laboratory results of creatinine (a), CPK (b), and troponin (c) during in-hospital stay.

Laboratory work-up showed deranged renal functions with blood urea nitrogen (BUN) 106 mg/dL, serum creatinine (Cr) of 5.32 mg/dL, and BUN : Cr ratio of 20.2. His urine osmolality was low (259 mosmol/L) and serum osmolality was high (290.1 mosmol/L) with urine/plasma osmolality ratio of 0.8 and urinary spot Na+ of 81 mmol/L. Urine analysis tested positive for pigmented casts. His serum creatinine phosphokinase (CPK) level was significantly high and has almost doubled after 6 hours (Table 1 and Figure 1(b)).

**Figure 2:**
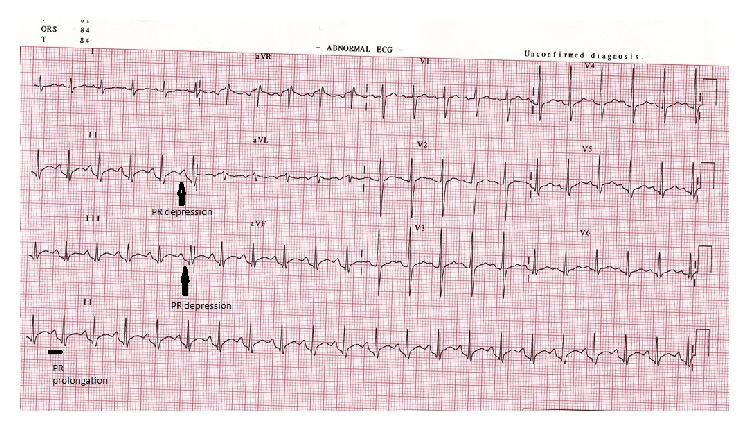
12 lead ECG at the time of presentation showing sinus tachycardia, prolonged PR interval (220 msec), and PR segment depression (4 mm).

**Figure 3:**
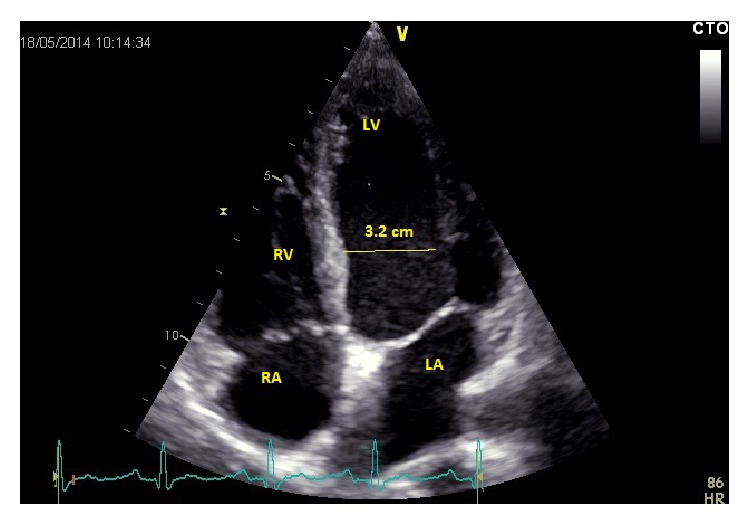
Transthoracic two-dimensional echocardiography showing normal sized ventricular cavities (LV dimension in systole is 3.2 cm) and normal heart valves. LV: left ventricle, LA: left atrium, RA: right atrium, and RV: right ventricle.

His ECG showed sinus tachycardia and the PR interval was strikingly depressed and prolonged (Figure 2). His cardiac markers were markedly elevated (Table 1 and Figure 1(c)). An emergency bedside echocardiography was done to look for evidence of acute myocardial infarction given the patient's presentation with shock and elevated cardiac marker. His echo showed generalized hypokinesia of the left ventricle wall with preserved left ventricular systolic function and normal cavity size. There were no regional wall motion abnormalities (Figure 3).

The patient got admitted to the intensive care unit. He was resuscitated with intravenous fluid therapy along with intravenous antibiotics. A central venous catheter and a urinary catheter were inserted to monitor the fluid balance and delivering IV medications. Patient was started, after sending cultures, on empirical antibiotic therapy with Metronidazole, Meropenem, and Levofloxacin. He needed 17 liters of fluid to maintain his hemodynamics.

His stool analysis showed numerous pus cells, and his subsequent stool culture was positive for* Salmonella enteritidis* and negative for Rotavirus Ag, Adenovirus, and* Clostridium difficile* toxin antigen.* Salmonella enteritidis was* also grown in blood cultures. Arterial blood gas (ABG) analysis showed significant anion gap metabolic acidosis with calculated serum anion gap of 16 which was significantly high.

**Figure 4:**
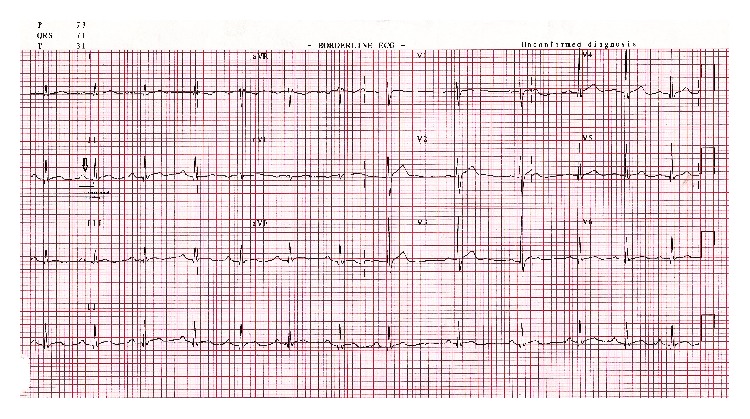
12 lead ECG showing normalization of PR interval (200 msec) and PR segment changes after initiation of treatment with intravenous antibiotics and fluids.

The patient responded well to the medical management. As his cardiac markers showed a downward trend, no additional cardiac medications were given during his in-hospital stay. His renal parameters, CPK, and cardiac enzymes showed a downward trend during the next 4 days. He was discharged home on the fifth day. Two weeks later, follow-up echocardiography and ECG were normal and cardiac markers showed normalization (Figure 4). He was advised to do regular checkups at the cardiology clinic looking for long-term complications such as cardiomyopathy. To date, the patient is doing well and leading a normal life.

## 3. Discussion

*Salmonella* is an enteric gram-negative pathogen and a common cause of self-limiting foodborne diseases. Death is very rare (1%) in immunocompetent patients. Myocarditis and rhabdomyolysis have been very rarely reported in patients with salmonella gastroenteritis. Patients with immunocompromised state may develop complications similar to those with typhoid fever.

Myocarditis was identified in 9% of postmortem examinations of patients who died from salmonella infection [4]. ECG abnormalities with widespread ST, T changes are characteristic of myocarditis [5]. Echocardiographic abnormalities of global hypokinesia differentiate myocarditis from other causes of cardiac enzyme elevation [6]. Pancarditis is a rarely reported complication with salmonella species [7]. Endomyocardial biopsy, the gold-standard test, has its own pitfalls and complications; hence, it is only recommended when the diagnosis could not be confirmed. Cardiac MRI was an option for proving myocarditis but it was not considered at this point, as the priority was to stabilize the patient and treat the infection. The patient showed sustained clinical improvement and his troponin levels declined gradually after starting him on IV antibiotics.

Our case was a young, immunocompetent man with no risk factors for coronary artery disease. The ECG findings of PR segment depression and first-degree AV block were consistent with a diagnosis of myocarditis rather than myocardial infarction. The rise of cardiac troponin-I indicates myocardial damage and the complete restoration of ECG and echo abnormalities was consistent with heart inflammation.

Rhabdomyolysis could be caused by toxin release, direct bacterial invasion, tissue hypoxia, or metabolic abnormalities. It is commonly manifested as muscle aches and dark-coloured urine. In more severe cases it can lead to acute kidney injury which is the cause of death in 8% of cases [8]. CPK levels that are 5–10 times the normal value are diagnostic for rhabdomyolysis with 100% sensitivity. Gastrointestinal loss of potassium could have contributed to the lack of hyperkalemia in our patient. The etiology of acute renal failure in this case was due to a combination of prerenal secondary to severe dehydration and renal secondary to tubular damage caused by hypoperfusion and the toxic effect of myoglobin.

Myocarditis and rhabdomyolysis can be serious complication of invasive salmonella infection. Proposed mechanisms of these complications include toxin release, direct invasion of the muscles, and altered metabolism in the muscle fibers. Salmonella infection should be considered in the differential diagnosis of patients presenting with myocarditis or rhabdomyolysis, as early institution of appropriate treatment is associated with good outcome [9].

## Figures and Tables

**Table 1:** The values of renal parameters, creatinine phosphokinase (CPK), C-reactive protein (CRP), and cardiac markers during the in-hospital stay of a patient with salmonella gastroenteritis complicated with rhabdomyolysis, acute kidney failure, and presumed myocarditis.

Blood test	Day 1	Day 1 after (6 hrs)	Day 2	Day 3	Day 5
Troponin-I ng/mL	22.47	13.09	5.38	1.0	0.47

CK-MB ng/mL	61.4	95.4	43.7	15	6

Urea mg/dL	106	93	35	23	32

S. creatinine mg/dL	5.23	3.78	1.30	1.0	0.8

Potassium K+ mmol/L	3.6	4.6	4.7	4.0	2.9

Sodium Na+ mmol/L	136	136	136	140	141

CPK-total (30–200) SI U/L, male	2644	4572	3408	2014	336

CRP mg/L	259.3	287.3	100	43	15.3

Serum calcium (8.4–10.2) mg/dL	8.0	8.4	—	—	9.2

Serum phosphorus mg/dL 2.3–4.7	5.8	—	—	—	4.5
